# Safety and efficacy of transcatheter arterial embolization in renal angiomyolipomas: a systematic review and meta-analysis

**DOI:** 10.1186/s12882-024-03893-4

**Published:** 2025-03-31

**Authors:** Shahid Miangul, Karen Smayra, Liranne Bitton, Reem B. Zalloum, Nathanael Q. E. Yap, Baraa Saad, Rangish Yuvaraj, Marios Alogakos, Hayato Nakanishi, Christian A. Than, Aneeta Parthipun

**Affiliations:** 1 St George’s University of London, London, SW17 0RE UK; 2 University of Nicosia Medical School, University of Nicosia, Nicosia, 2417 Cyprus; 3 College of Liberal Arts and Sciences, University of Illinois at Chicago (UIC), Chicago, 60607 USA; 4 Baylor Scott & White Allsaints Medical Center, Fort Worth, Texas, TX 76104 USA; 5 School of Biomedical Sciences, The University of Queensland, St Lucia, Brisbane, 4072 Australia; 6 Cambridge University Hospitals NHS Foundation Trust, School of Biomedical Sciences, Cambridge University Hospitals NHS Foundation Trust, School of Biomedical Sciences, Hills Road, Cambridge, Cambridgeshire, CB2 0QQ UK; 7 Interventional Radiology, Guy’s, St Thomas’ and Evelina Children’s Hospital, London, SE17EH UK

**Keywords:** Renal angiomyolipoma, Transcatheter arterial embolization, Tumour size reduction, Technical success, Meta-analysis

## Abstract

**Purpose:**

Transcatheter arterial embolization (TAE) is increasingly utilized in the management of renal angiomyolipomas (AML). However, the growth in its application has not been matched by a proportional increase in the level of evidence validating its safety and efficacy. This meta-analysis aims to evaluate TAE in managing renal AMLs by examining the effects of tumour size reduction and technical success.

**Methods:**

A literature search of several databases was conducted from inception to July 2023. Eligible studies reported adult patients (≥ 18 years old) who underwent TAE for renal AML. The pooled proportions were analyzed using a random-effects model. This review was registered in PROSPERO (CRD42023441331).

**Results:**

A total of 32 studies comprising 1087 patients were included. The average preoperative tumour size across renal AMLs was 8.79 cm (95% CI: 7.64, 9.93; *I*^*2*^ = 97%) and the post-operative tumour size was 6.47 cm (95% CI: 5.44, 7.50; *I*^*2*^ = 98%). The mean decrease in tumour size was 1.85 cm (95% CI: 1.69, 2.00; *I*^*2*^ = 23%), whereas the mean reduction in tumour size percentage was 43.30% (95% CI: 34.30, 52.30; *I*^*2*^ = 98%). The technical success rate from 1059 embolizations was 95.70% (95% CI: 0.94, 0.97; *I*^*2*^ = 44%), with 65 procedural failures reported.

**Conclusion:**

This meta-analysis provides insights into the efficacy of TAE for renal AMLs, emphasizing notable tumour size reduction and a high technical success rate for selected patients. Despite identified biases, the findings support TAE as a valuable intervention, warranting further research to refine safety profiles and optimize patient outcomes.

**Supplementary Information:**

The online version contains supplementary material available at 10.1186/s12882-024-03893-4.

## Background

Renal angiomyolipoma (AML), constituting the most prevalent benign renal tumour, accounts for 0.2–0.6% of all renal masses, displaying a female predilection exceeding 2:1 [1, 2]. While 80% of cases occur sporadically, the remaining 20% are associated with tuberous sclerosis complex (TSC) or pulmonary lymphangioleiomyomatosis (LAM) [3]. Typically, renal AMLs follow a benign course due to gradual growth with symptoms being manifested when the tumour size reaches 4 cm or greater, but their association with TSC leads to rapid growth [4]. AMLs are classically diagnosed by the presence of fat on ultrasonography and computed tomography (CT) [5], and magnetic resonance (MR) imaging could achieve 99% specificity, even in fat-poor AMLs [6]. Currently, the European Association of Urology and Canadian Urological Association recommend that asymptomatic renal AMLs of < 4 cm can be observed, while symptomatic or renal AMLs > 4 cm require treatment [7–9].

Traditionally, nephron sparring surgery has seen great success in the management of AMLs [10], however more minimally invasive approaches such as transcatheter arterial embolization (TAE) techniques have emerged and seen success since 1986 [11]. TAE is a sophisticated and targeted approach that selectively occludes the feeding arteries of the tumour and induces a controlled ischemic environment within the AML while minimizing collateral damage to the surrounding healthy renal tissue [12]. This nuanced technique showcases TAE as a valuable option for both acute scenarios involving hemorrhage and as a proactive measure to limit the vascular supply to renal AMLs, preventing their growth and spontaneous rupture.

Given the multifaceted nature of renal AMLs, characterized by a combination of thick-walled dysmorphic vessels, smooth muscle, and mature adipose tissue, the dysmorphic and tortuous nature of blood vessels within these tumours poses an inherent risk of aneurysm formation and subsequent hemorrhage. TAE strategically addresses this risk by providing a localized intervention that mitigates the potential for life-threatening bleeding episodes. The versatility of TAE positions it as an attractive choice for patients seeking therapeutic benefits while retaining crucial renal function.

In keeping with consensus among major guidelines, such as those from the CUA and EAU, which recommend intervention for AMLs based on factors including size and symptoms, the literature consistently supports the use of TAE, showing promising and convincing outcomes when indicated. This meta-analysis aims to evaluate the efficacy and safety of TAE in the management of renal AMLs, with a focused exploration on technical success rates, tumour size reduction, and post-procedural complication rates. This nuanced perspective seeks to expand upon promising findings in the literature, offering up-to-date guidance to clinicians for informed decisions customized to individual patient scenarios.

## Materials and methods

### Search strategy and data sources

This review followed the Preferred Reporting Items for Systematic Reviews and Meta-analyses (PRISMA) guidelines [13]. A comprehensive search of several databases from inception to July 26, 2023, was conducted with no language restriction. The databases included PubMed, EMBASE (Elsevier), CiNAHL, Cochrane Central Register of Controlled Trials, Cochrane Database of Systematic Reviews, Scopus, and Web of Science. The search strategy was designed and conducted by a medical reference librarian with input from the study’s principal investigator. Controlled vocabulary supplemented with keywords was used to search for studies on adult patients who underwent transarterial embolization for renal angiomyolipomas. The actual strategy listing all search terms used and how they are combined is available in Additional file 1. Additional references were sought through hand searches of Google scholar (search terms: renal angiomyolipoma; embolization; artificial; therapeutic, transarterial). This review was registered prospectively with PROSPERO (CRD42023441331). Although our initial PROSPERO registration specified the use of the Newcastle-Ottawa Scale (NOS) for assessing risk of bias, we subsequently adopted the ROBINS-I (Risk Of Bias In Non-randomized Studies - of Interventions) tool. This change was made to better align with the non-randomized nature of the included studies and to provide a more comprehensive assessment of bias specific to our study design. ROBINS-I offers a detailed evaluation across several domains of potential bias, which was crucial for the rigor of our meta-analysis.

### Eligibility criteria, quality assessment and study arms

Eligible studies for this meta-analysis were required to fulfill the following inclusion criteria: 1) Inclusion of adult patients (aged > 18 years) undergoing transcatheter arterial embolization for renal AML; 2) Reporting one of the primary outcomes, the first being outcomes related to tumor size reduction, including tumor percentage reduction, pre- and post-operative tumour size as well as pre- and post-operative tumour volume or 3) Reporting of outcomes related to technical success; 4) In cases where multiple studies were conducted by the same institution and/or authors, inclusion was based on the study of higher quality or the most recent publication. 5) Studies were excluded if they contained unpublished data, were published only in abstract form, or were non-full-length articles; 6) If it was impractical to extract or calculate appropriate data from published results; 7) If there was significant overlap between authors, centers, or patient cohorts across the published literature; 8) If the literature of interest was not available in English; 9) Exclusion of case reports, case series, review articles, and abstracts. Article screening and data extraction were carried out independently by four assessors (LB, RZ, RY, SM), with any discrepancies adjudicated by SM and discussed with co-authors as needed. In our study, we employed COVIDENCE to automate the abstract screening, full-text screening, and selection of included studies, which streamlined the review process and improved the efficiency and consistency of identifying relevant studies for our meta-analysis. The quality of each study was independently assessed by three authors (RZ, LB, BS) using the ROBINS-1 quality assessment scale. Any discrepancies in quality determination were resolved through discussion with a third author until a consensus was reached (SM).

### Definitions and outcomes

Technical success and reduction of the tumour were the outcome variables of interest in this meta-analysis. Technical success in TAE of renal AML was defined as the achievement of tumor vascular occlusion, characterized by the cessation of flow in the target vessels supplying the AML and the absence of tumor staining following contrast administration. The reduction in tumour size was evaluated under different units of measurement: cm, cm^2^, cm^3^, mL and % reduction. Additionally, the discreet values pertaining to preoperative and postoperative tumour sizes were recorded.

### Statistical analysis

The pooled means of continuous variables and rates of binary variables were pooled using the generic inverse variance method of DerSimonian and Laird (1986), which assigned the weight of each study based on its variance [14]. The heterogeneity of effect size estimates across the studies was quantified using the Q statistic and the I^2^ index (*P* < 0.10 was considered significant) [15]. A value of I^2^ of 0–25% indicates minimal heterogeneity, 26–50% moderate heterogeneity, and 51–100% high heterogeneity. Furthermore, a leave-one-out sensitivity analysis was conducted to assess each study’s influence on the pooled estimate by omitting one study at a time and recalculating the combined estimates for the remaining studies. If mean and standard deviation (SD) were unavailable, median was converted to mean using the formulas from the Cochrane Handbook for Systematic Reviews of Interventions [16]. Authors were contacted three times to obtain any relevant additional information that was omitted in published articles. Publication bias was assessed using a funnel plot [17]. Data analysis was performed using Open Meta analyst software (CEBM, Brown University, Providence, Rhode Island, USA).

## Results

### Study selection and patient characteristics

The initial literature search of electronic databases resulted in a total of 1065 studies. After removing duplicates, 1017 articles were screened for inclusion and exclusion criteria and 85 full texts were retained for full-text review. Thirty-two studies met the criteria and were included in this quantitative meta-analysis, as depicted in Table 1. All the eligible studies were retrospective studies, with the exception of Abouelkheir et al. (2022), which was a prospective single centre study [18]. The date of publication ranged between 1997 and 2023. Overall, there was a total of 1087 patients, of which 772 (71.02%) were female and 315 (28.98%) were male. The weighted mean age of patients was reported to be 46.25 ± 15.17 years. The weighted mean follow-up of patients was reported to be 34.72 ± 45.66 months. The study selection process is illustrated in the PRISMA flow chart in Additional file 2.

**Table 1:** Baseline characteristics of included studies

Study	Year	Country	Number of Participants (*n*)	Gender Male/Female (*n*)	Age, Mean ± SD years	Follow up, Mean ± SD months
**Abouelkheir et al. ** [18]	2022	Egypt	33	9/24	38.3 ± 13.9	6.9 ± 3.4
**Ahmadov et al.** [19]	2022	Turkey	42	37/5	43.5 ± 15.3	26.5 ± 25.7
**Anis et al.** [20]	2020	Palestine	74	10/64	52.0 ± 15.5	132.4 ± 52.9
**Baba et al.** [21]	2014	Japan	11	3/8	55.1 ± 13.8	NR
**Bardin et al.** [22]	2017	France	23	5/18	52.0 ± 19.0	20.5 ± 13.9
**Bishay et al.** [23]	2010	USA	16	2/14	41.2 ± 15.0	29.0 ± 19.8
**Chaiyasoot et al.** [24]	2021	Thailand	21	5/16	47.0 ± 11.5	41.3 ± 13.5
**Chan et al.** [25]	2011	China	27	6/21	46.3 ± 10.8	85.3 ± 19.9
**Chan Park et al.** [26]	2021	South Korea	15	1/14	52.0 ± 14.8	NR
**Chapman et al.** [27]	2021	New Zealand	20	5/15	54.0 ± 16.0	42.0 ± 42.5
**Chatziioannou et al.** [28]	2012	Greece	10	2/8	36.0 ± 12.2	9.0 ± 2.3
**Combes et al.** [29]	2023	Australia	26	6/20	47.2 ± 12.7	5.3 ± 14.8
**El Rafei et al.** [30]	2015	France	24	2/22	43.0 ± 12.0	42.0 ± 36
**Farg et al.** [31]	2022	Egypt	148	105/43	45.0 ± 15.0	NR
**Han et al.** [32]	1997	South Korea	14	2/12	50.0 ± 12.8	33.0 ± NR
**Hocquelet et al.** [33]	2014	France	19	1/18	47.2 ± 21.0	28.0 ± NR
**Hongyo et al.** [34]	2020	Japan	44	12/32	34.5 ± 15.1	49.9 ± 36.3
**Kothary et al.** [35]	2005	USA	19	2/17	48.5 ± 12.0	51.5 ± 31.5
**Lee et al.** [36]	2021	NR	119	22/97	45.4 ± 13.8	NR
**Lee et al.** [37]	2009	Taiwan	11	2/9	49.0 ± 12.5	28.3 ± 23.1
**Lin et al.** [38]	2018	China	45	9/36	38.6 ± 12.5	14.0 ± 29.8
**Nozadze et al.** [39]	2022	NR	49	10/39	50.0 ± 9.5	55.2 ± 11.1
**Planche et al.** [40]	2011	France	30	5/25	44.0 ± 13.0	20.5 ± 11.9
**Prigent et al.** [41]	2021	France	24	2/22	51.0 ± 20.8	15.0 ± 17.5
**Ramon et al.** [42]	2009	Palestine	42	5/37	51.0 ± 14.5	58.0 ± 36.3
**Rimon et al.** [43]	2006	Israel	17	4/13	55.0 ± 14.5	23.0 ± 14.0
**Rolland et al.** [44]	2023	NR	24	3/21	53.9 ± 16.2	NR
**Salık et al.** [45]	2019	Turkey	13	5/8	44.9 ± 14.6	6.0 ± NR
**Takebayashi et al.** [46]	2009	Japan	10	3/7	41.0 ± 6.5	26.4 ± 11.8
**Urbano et al.** [47]	2017	Spain	22	7/15	53.5 ± 12.5	36.7 ± 13.0
**Wang et al.** [48]	2017	NR	79	19/60	40.3 ± 14.8	35.9 ± NR
**Williams et al.** [49]	2006	USA	16	4/12	29.7 ± 9.3	16.0 ± 7.8

*n *number, *NR *not reported, *SD *standard deviation

### Risk of bias and quality assessment

The results of the quality assessment of the included studies are summarized in Additional file 3. The ROBINS-1 tool was used to systematically evaluate both the risk of bias and the overall quality of the thirty two selected studies. Eleven studies were categorized as demonstrating a moderate risk of bias [18, 19, 25, 29, 31, 33, 34, 38, 40, 49, 50]. On the other hand, twenty studies were classified as displaying serious risk of bias [20, 20–24, 26–28, 30, 32, 35–37, 39, 41, 42, 44–46], and one study was critical risk of bias [47]. Nonetheless, all the studies included were deemed adequate within the selection domain.

### Clinical characteristics

**Fig. 1:**
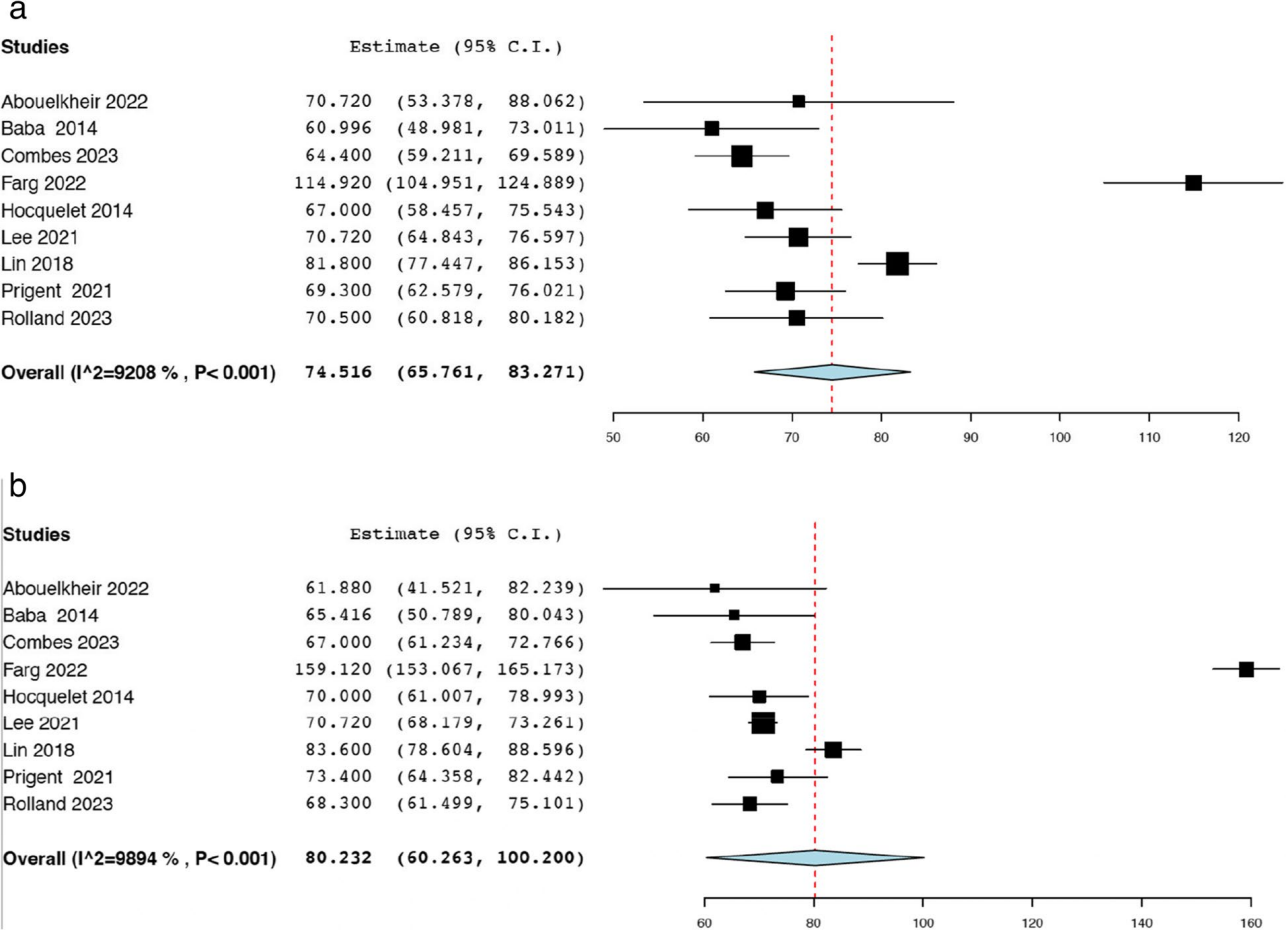
**a** Pooled pre-operative serum creatinine (μmol/L). **b **Pooled post-operative serum creatinine (μmol/L)

Upon diagnosis of renal AML, 414 patients were reported to be symptomatic across twenty-two studies [18–21, 24, 25, 27, 29, 30, 32, 34–38, 40–44, 47, 50]. From fifteen studies, 100 patients underwent an emergency procedure from initial presentation [18–21, 23, 25–27, 29, 33, 34, 39, 42, 44, 46] and from nine studies 167 patients had an incidental discovery of the diagnosis [25, 27, 31, 36, 37, 40, 42, 43, 50]. Furthermore, 286 cases were reported to receive prophylactic intervention for their AML according to seven studies [18, 20, 23, 25, 27, 38, 46]. Upon presentation, 130 patients displayed symptoms of bleeding/haemorrhage across sixteen of the included studies [18–21, 29, 32, 35, 37, 39–44, 47, 49], 235 experienced pain according to twenty studies [18, 20, 22, 23, 27, 29–32, 34, 35, 37, 39–43, 47, 49, 50], 172 reported haematuria from twenty-two studies [18, 20, 22, 23, 27, 29, 31–35, 37, 39–44, 46, 47, 49, 50] and 97 had aneurysms according to eight studies [18–20, 33, 34, 38, 39, 42]. Nine studies reported the serum creatinine levels before TAE, encompassing a total of 449 patients with renal AMLs [18, 26, 29, 31, 33, 37–39, 42]. The pooled average pre-operative serum creatinine level was calculated to be 74.52 µmol/L (95% CI: 65.76, 83.27; *I*^*2*^ = 92.08%) (Fig. 1a). Nine studies provided data on serum creatinine levels after the intervention, involving a total of 449 patients [18, 26, 29, 31, 33, 37–39, 42]. The pooled postoperative serum creatinine level was 80.23 µmol/L (95% CI: 60.26, 100.20; *I*^*2*^ = 98.94%) (Fig. 1b). The data pertaining to clinical characteristics of included studies is summarized in Table 2.

**Table 2:** Clinical characteristics

Study	Clinical Presentation (*n*)							Renal Function Pre-TAE, Mean ± SD mL/min/1.73 m^2^	Serum Creatinine Pre-TAE, Mean ± SD µmol/L
	Symptomatic	Emergency	Prophylactic	Incidental Discovery	Haemorrhage / Bleeding	Pain	Haematuria		
**Abouelkheir et al.**	30	29	3	NR	30	35	12	NR	70.7 ± 50.8
**Ahmadov et al.**	27	2	NR	NR	9	NR	NR	NR	NR
**Anis et al.**	71	NR	NR	34	18	14	1	NR	NR
**Baba et al.**	NR	1	NR	NR	NR	NR	NR	81.8 ± 25.3	60.9 ± 20.3
**Bardin et al.**	17	6	17	NR	6	7	4	78.0 ± 21.0	NR
**Bishay et al.**	NR	NR	NR	NR	NR	3	2	NR	79.6 ± 15.5
**Chaiyasoot et al.**	NR	9	12	NR	NR	14	1	NR	NR
**Chan et al.**	NR	15	13	NR	NR	NR	NR	NR	NR
**Chan Park et al.**	9	NR	NR	NR	NR	NR	NR	80.0 ± 22.9	NR
**Chapman et al.**	4	4	NR	NR	2	NR	NR	NR	76.0 ± NR
**Chatziioannou et al.**	7	3	7	3	NR	4	3	NR	NR
**Combes et al.**	11	5	NR	NR	4	9	2	NR	64.4 ± 13.5
**El Rafei et al.**	NR	NR	NR	NR	NR	NR	NR	NR	NR
**Farg et al.**	NR	NR	NR	1	NR	34	109	NR	114.9 ± 61.9
**Han et al.**	14	NR	NR	NR	NR	11	NR	NR	NR
**Hocquelet et al.**	NR	NR	NR	NR	NR	NR	NR	NR	67.0 ± 19.0
**Hongyo et al.**	6	2	NR	NR	NR	3	3	NR	NR
**Kothary et al.**	16	NR	NR	NR	7	6	3	NR	NR
**Lee et al.**	25	NR	NR	26	12	1	NR	NR	70.7 ± 32.7
**Lee et al.**	9	NR	NR	NR	5	9	3	NR	NR
**Lin et al.**	25	NR	45	NR	NR	NR	NR	NR	81.8 ± 14.9
**Nozadze et al.**	13	NR	NR	36	NR	NR	NR	85.0 ± 6.6	NR
**Planche et al.**	7	NR	NR	23	1	7	NR	NR	81.0 ± NR
**Prigent et al.**	NR	4	20	NR	4	13	2	NR	69.3 ± 16.8
**Ramon et al.**	41	NR	NR	NR	10	8	3	NR	NR
**Rimon et al.**	13	NR	NR	NR	8	4	1	NR	NR
**Rolland et al.**	8	4	NR	13	4	4	2	NR	70.5 ± 24.2
**Salık et al.**	13	6	NR	NR	6	NR	7	NR	NR
**Takebayashi et al.**	NR	NR	NR	NR	NR	NR	NR	NR	NR
**Urbano et al.**	NR	10	17	NR	NR	NR	2	NR	NR
**Wang et al.**	48	NR	NR	31	NR	45	10	NR	NR
**Williams et al.**	NR	NR	NR	NR	4	4	2	95.8 ± 22.0	NR

*Min* minute, *mL* milliliters, *n* number, *NR* not reported, *SD* standard deviation, *TAE* transarterial embolization

### Pre-operative tumour characteristics

Co-morbidity with TSC was reported in 232 patients across twenty-four of the included studies [18–21, 23, 26–29, 32–43, 46, 47, 50]. The location of AML lesions was reported in sixteen of the studies [18–21, 25, 26, 29–31, 37, 39, 41–43, 45, 50]. 346 of the lesions were described as right-sided and 314 were left-sided. Additionally, a total of 223 patients belonging to twenty-two studies were reported to have bilateral lesions [18, 20–27, 30–32, 34, 36–39, 41, 43, 45, 46, 50]. Additionally, six of the studies did not report the location of the lesions [28, 33, 40, 44, 47, 49]. Four studies described a total of 153 exophytic lesions [37, 39, 42, 46] and three studies reported 18 central lesions [39, 45, 46]. Furthermore, 142 lesions reportedly presented as a single mass according to eleven studies [18, 20, 23, 25–28, 34, 38, 46, 50] and 197 were identified within clusters of multiple masses as reported by thirteen of the studies [18, 20, 23–28, 34, 38, 39, 46, 50]. Overall, a total of 979 renal AMLs were reported across all of the included studies except four [21, 38, 43, 50]. The data corresponding to pre-operative tumour characteristics is available in Table 3.

**Table 3:** Pre-operative tumour characteristics

Study	Tuberous Sclerosis (*n*)	Side of AML (*n*)			Location of AML (*n*)				Number of Masses (*n*)		Total Number of Renal AML (*n*)	Tumour Enhancement (*n*)	
		Right	Left	Bilateral	Central	Exophytic	Hilar	Intraparenchymatous	Single	Multiple		≤ 50%	> 50%
**Abouelkheir et al.**	12	9	8	16	NR	NR	NR	NR	15	18	36	NR	NR
**Ahmadov et al.**	14	25	NR	NR	NR	NR	NR	NR	NR	NR	48	NR	NR
**Anis et al.**	13	44	30	NR	NR	NR	NR	NR	NR	NR	NR	NR	NR
**Baba et al.**	0	9	2	0	NR	NR	NR	NR	11	NR	11	NR	NR
**Bardin et al.**	17	18	16	9	NR	NR	NR	NR	11	12	34	NR	NR
**Bishay et al.**	NR	NR	NR	6	NR	NR	NR	NR	NR	NR	23	NR	NR
**Chaiyasoot et al.**	4	NR	NR	13	NR	NR	NR	NR	12	9	25	NR	NR
**Chan et al.**	NR	14	14	8	NR	NR	NR	NR	17	11	28	NR	NR
**Chan Park et al.**	NR	NR	NR	4	NR	NR	NR	NR	NR	6	15	14	1
**Chapman et al.**	2	9	12	4	NR	NR	NR	NR	NR	NR	NR	NR	NR
**Chatziioannou et al.**	2	NR	NR	2	NR	NR	NR	NR	2	8	12	NR	NR
**Combes et al.**	7	11	19	NR	NR	NR	NR	NR	NR	NR	30	NR	NR
**El Rafei et al.**	9	NR	NR	NR	NR	NR	NR	NR	11	13	30	NR	24
**Farg et al.**	NR	64	82	2	NR	NR	NR	NR	NR	NR	148	NR	NR
**Han et al.**	NR	NR	6	2	NR	NR	NR	NR	NR	NR	16	NR	NR
**Hocquelet et al.**	7	NR	NR	NR	NR	NR	NR	NR	NR	NR	39	22	14
**Hongyo et al.**	29	NR	NR	25	NR	NR	NR	NR	14	16	50	NR	NR
**Kothary et al.**	10	NR	NR	10	NR	NR	NR	NR	NR	NR	30	NR	NR
**Lee et al.**	8	61	58	0	NR	85	16	NR	NR	NR	119	46	73
**Lee et al.**	4	NR	NR	NR	NR	NR	NR	NR	NR	NR	11	NR	NR
**Lin et al.**	9	NR	NR	30	NR	NR	NR	NR	14	31	NR	29	16
**Nozadze et al.**	20	NR	NR	22	NR	NR	NR	NR	NR	NR	53	NR	NR
**Planche et al.**	18	NR	NR	NR	NR	NR	NR	NR	NR	NR	34	13	11
**Prigent et al.**	5	14	13	7	2	25	NR	NR	NR	7	27	NR	NR
**Ramon et al.**	8	27	21	8	NR	NR	NR	NR	NR	NR	48	NR	NR
**Rimon et al.**	5	NR	NR	NR	NR	NR	NR	NR	NR	NR	18	NR	NR
**Rolland et al.**	1	11	14	NR	NR	24	NR	1	NR	NR	25	NR	NR
**Salık et al.**	NR	NR	NR	NR	NR	NR	NR	NR	NR	NR	14	NR	NR
**Takebayashi et al.**	NR	10	NR	NR	10	NR	NR	NR	N	NR	10	5	5
**Urbano et al.**	6	NR	NR	9	6	19	NR	NR	12	10	25	NR	NR
**Wang et al.**	22	14	19	46	NR	NR	NR	NR	23	56	NR	NR	NR
**Williams et al.**	NR	NR	NR	NR	NR	NR	NR	NR	NR	NR	20	NR	NR

*n *number, *NR *not reported, *SD *standard deviation, *AML* Angiomyolipoma

### Tumour-related outcomes

**Fig. 2:**
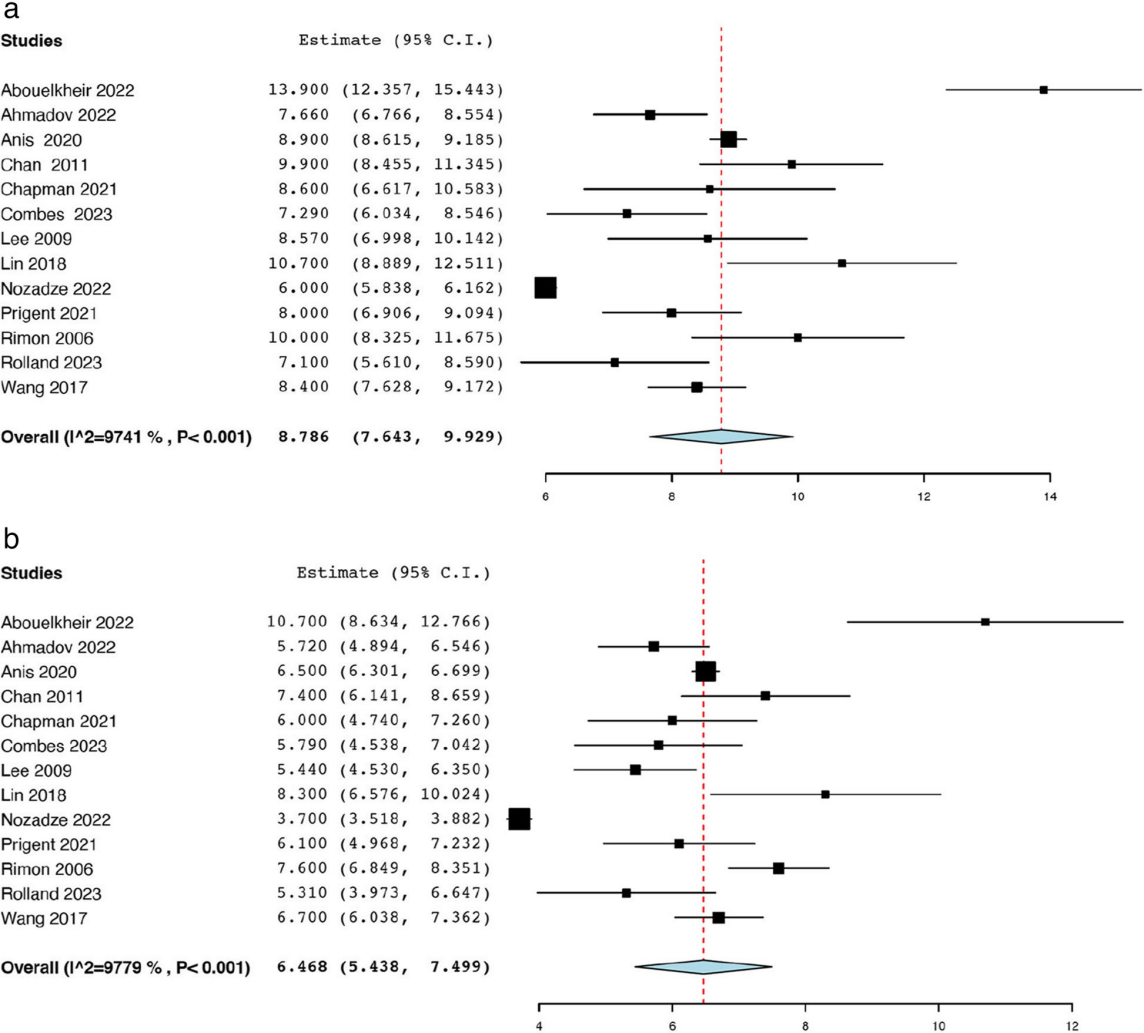
a Pooled mean pre-operative tumour size (cm). **b **Pooled mean post-operative tumour size (cm)

**Fig. 3:**
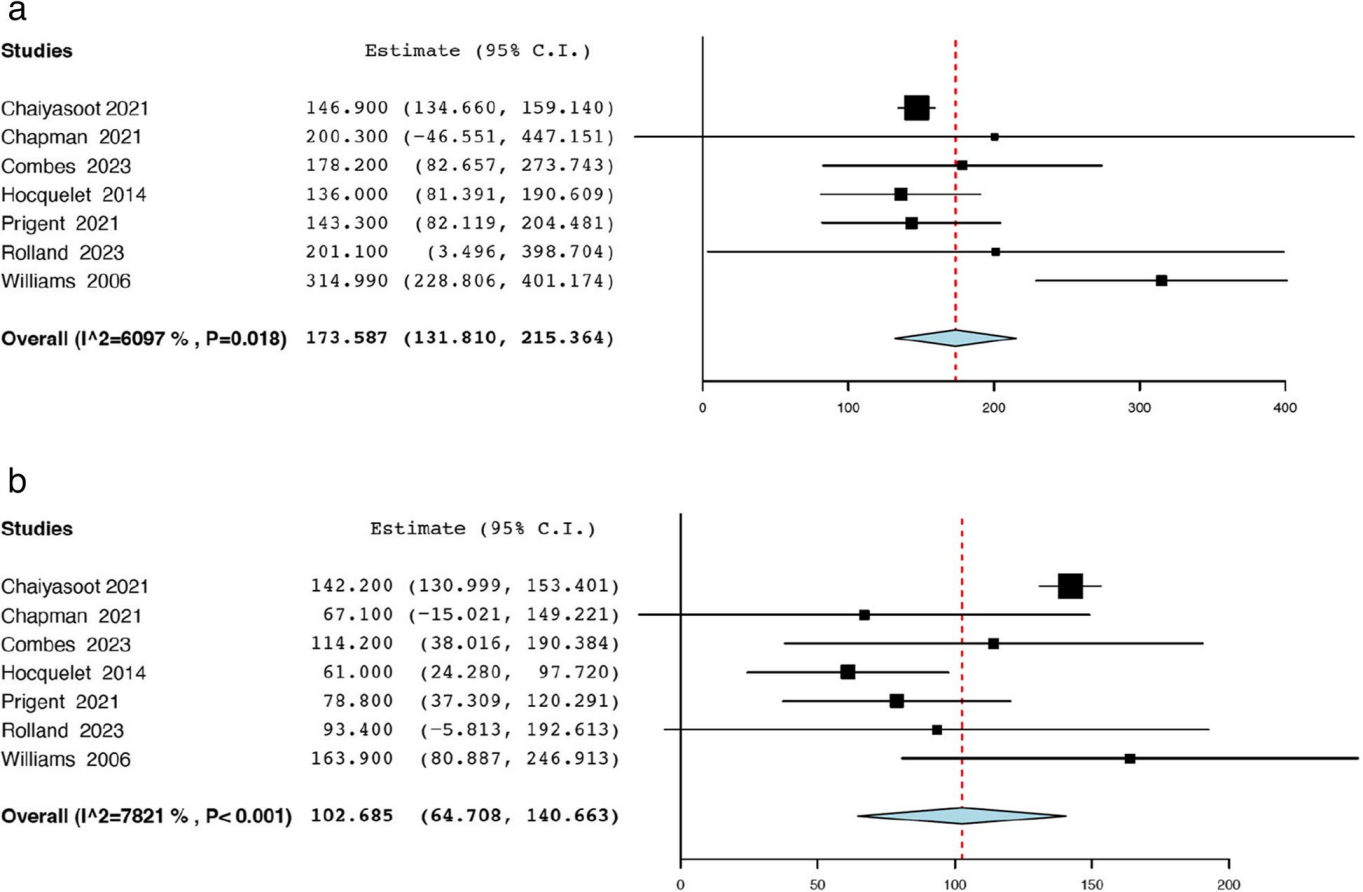
**a **Pooled mean pre-operative tumour volume (mL). **b **Pooled mean post-operative tumour volume (mL)

**Fig. 4:**
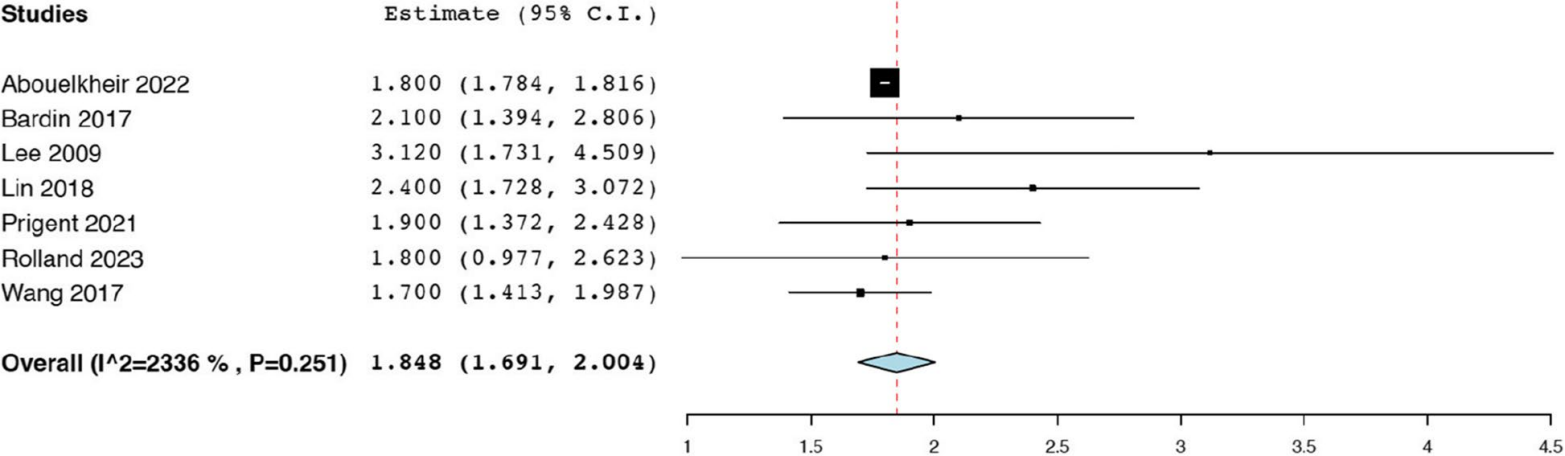
Pooled mean decrease in tumour size (cm)

**Fig. 5:**
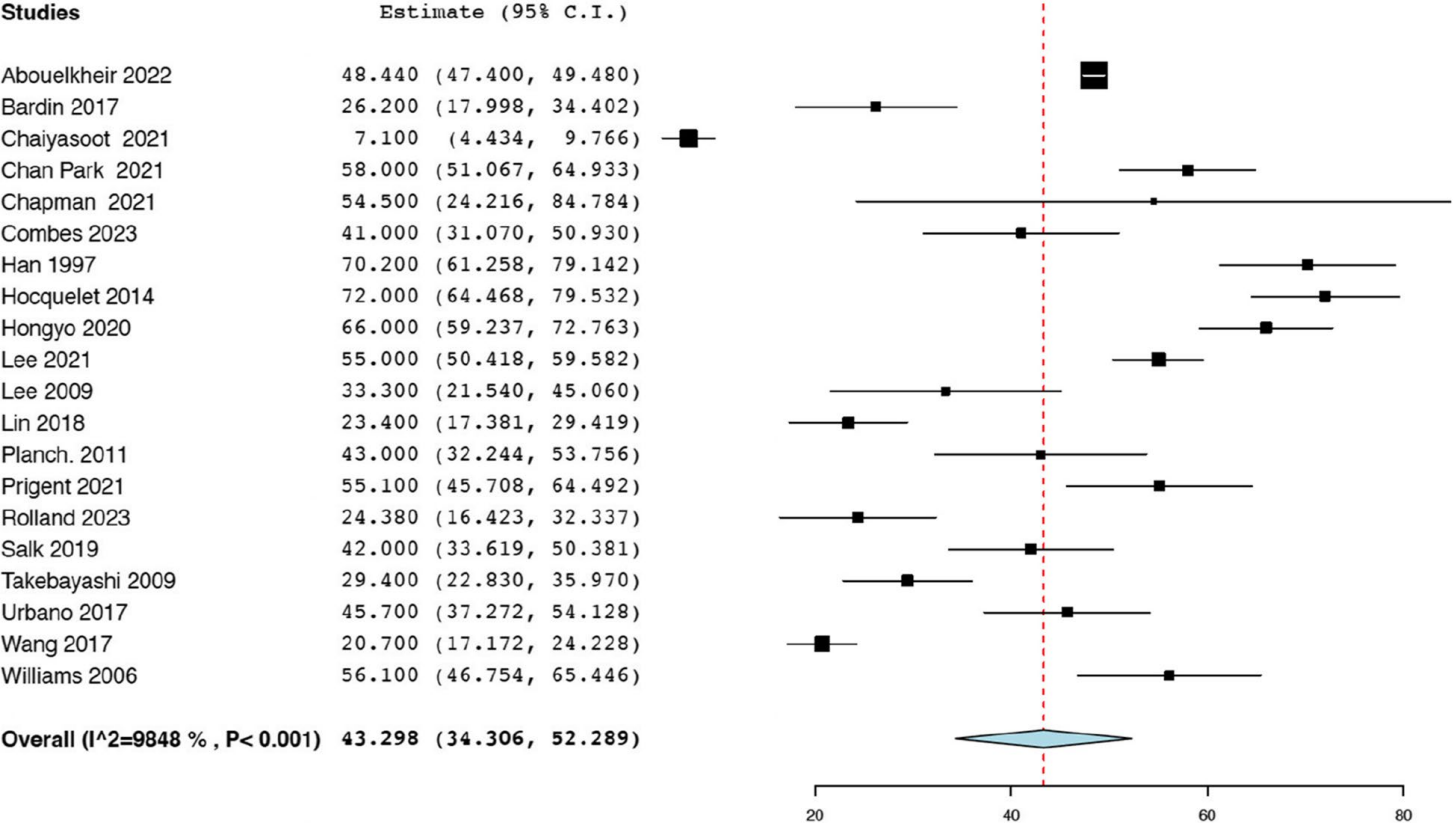
Pooled mean percentage reduction in tumour size (%)

Thirteen studies reported the initial tumour size before TAE from a total of 494 renal AMLs [18, 19, 21, 25, 29, 35, 36, 38, 39, 42, 43, 47, 50]. The analysis revealed that the average pooled pre-operative tumour size was 8.79 cm (95% CI: 7.64, 9.93; *I*^*2*^ = 97.41%) **(**Fig. 2a).Thirteen studies reported the post-operative tumour size after TAE from a total of 494 renal AMLs [18, 19, 21, 25, 29, 35, 36, 38, 39, 42, 43, 47, 50], and the pooled mean post-operative tumour size was 6.47 cm (95% CI: 5.44, 7.50; *I*^*2*^ = 97.79%) (Fig. 2b). Seven studies reported the mean tumour volume before TAE from a total of 186 renal AMLs [21, 23, 29, 33, 39, 42, 49]. The pooled average pre-operative tumour volume of 173.59 mL (95% CI: 131.81, 215.36; *I*^*2*^ = 60.97%) **(**Fig. 3a). Seven studies reported the mean tumour volume after TAE from a total of 186 renal AMLs [21, 23, 29, 33, 39, 42, 49]. The pooled average post-operative tumour volume was 102.69 mL (95% CI: 64.71, 140.66; *I*^*2*^ = 78.21%) (Fig. 3b). The data pertaining to post-procedural outcomes is detailed in Table 4. Seven studies reported the mean decrease in tumour size post-TAE from a total of 257 renal AMLs [18, 20, 35, 38, 39, 42, 50]. As such the pooled mean reduction in AML size was 1.85 cm (95% CI: 1.69, 2.00; *I*^*2*^ = 23.36%) **(**Fig. 4). Twenty studies reported the mean percentage reduction in tumour size after TAE from a total of 674 renal AMLs [18, 20, 21, 23, 24, 29, 30, 33–35, 37–40, 42, 44–46, 49, 50]. As such the pooled mean percentage reduction in AML size was 43.30% (95% CI: 34.31, 52.29 *I*^*2*^ = 98.48%) (Fig. 5).

**Table 4:** Tumour outcomes

Study	Pre-operative Tumour Size, Mean ± SD cm	Post-operative Tumour Size, Mean ± SD cm	Pre-operative Tumour Volume, Mean ± SD mL	Post-operative Tumour Volume, Mean ± SD mL	Reduction in Tumour Size, Mean ± SD %
**Abouelkheir et al.**	13.9 ± 4.7	10.7 ± 6.3	NR	NR	48.4 ± 3.2
**Ahmadov et al.**	7.7 ± 3.2	5.7 ± 2.9	NR	NR	NR
**Anis et al.**	8.9 ± 1.2	6.5 ± 0.9	NR	NR	27.0 ± NR
**Baba et al.**	NR	NR	NR	NR	NR
**Bardin et al.**	8.9 ± 5.0	NR	NR	NR	26.2 ± 24.4
**Bishay et al.**	15.0 ± 3.8	13.8 ± NR	NR	NR	NR
**Chaiyasoot et al.**	8.9 ± 3.7	NR	146.9 ± 31.2	142.2 ± 28.6	7.1 ± 6.8
**Chan et al.**	9.9 ± 3.9	7.4 ± 3.4	NR	NR	NR
**Chan Park et al.**	9.8 ± 4.1	NR	NR	NR	58.0 ± 13.7
**Chapman et al.**	8.6 ± 4.5	6.0 ± 2.9	200.3 ± 563.3	67.1 ± 187.4	54.5 ± 69.1
**Chatziioannou et al.**	8.2 ± 2.1	NR	NR	NR	NR
**Combes et al.**	7.3 ± 3.5	5.8 ± 3.5	178.2 ± 267.0	114.2 ± 212.9	41.0 ± 27.8
**El Rafei et al.**	7.0 ± 3.0	NR	NR	NR	60.0 ± NR
**Farg et al.**	NR	NR	NR	NR	NR
**Han et al.**	NR	NR	NR	NR	70.2 ± 18.3
**Hocquelet et al.**	7.0 ± NR	4.5 ± NR	136.0 ± 174.0	61.0 ± 117.0	72.0 ± 24.0
**Hongyo et al.**	NR	NR	NR	NR	66.0 ± 24.4
**Kothary et al.**	NR	NR	NR	NR	NR
**Lee et al.**	NR	NR	NR	NR	55.0 ± 25.5
**Lee et al.**	8.6 ± 2.7	5.4 ± 1.5	NR	NR	33.3 ± 19.9
**Lin et al.**	10.7 ± 6.2	8.3 ± 5.9	NR	NR	23.4 ± 20.6
**Nozadze et al.**	6.0 ± 0.6	3.7 ± 0.7	NR	NR	NR
**Planche et al.**	8.2 ± 3.7	NR	NR	NR	43.0 ± 32.0
**Prigent et al.**	8.0 ± 2.9	6.1 ± 3.0	143.3 ± 162.2	78.8 ± 110.0	55.1 ± 24.9
**Ramon et al.**	10.3 ± 4.4	NR	NR	NR	NR
**Rimon et al.**	10.0 ± 3.6	7.6 ± 1.6	NR	NR	24.0 ± NR
**Rolland et al.**	70.9 ± 38.0	53.1 ± 34.1	NR	NR	24.4 ± 20.3
**Salık et al.**	NR	NR	429.0 ± 318.5	NR	42.0 ± 16.0
**Takebayashi et al.**	7.0 ± 2.0	NR	NR	NR	29.4 ± 10.6
**Urbano et al.**	6.7 ± 2.4	NR	NR	NR	45.7 ± 21.5
**Wang et al.**	8.4 ± 3.5	6.7 ± 3.0	NR	NR	20.7 ± 16.0
**Williams et al.**	NR	NR	NR	NR	56.1 ± 21.3

*AML *Angiomyolipoma, *cm *centimeters, *mL* milliliters, *n *number, *NR *not reported, *SD *standard deviation

### Peri-operative outcomes

**Fig. 6:**
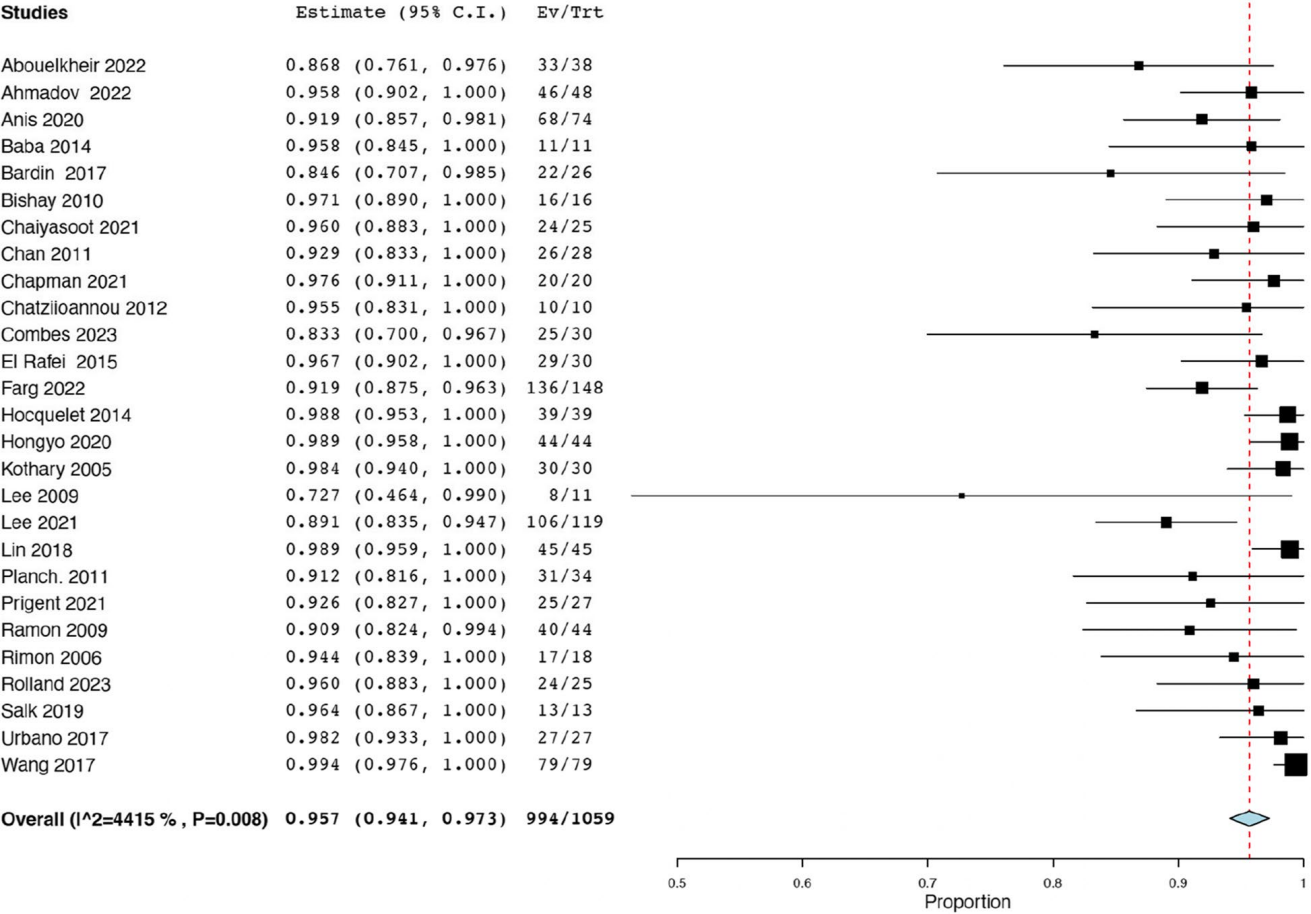
Pooled technical success rate (%)

Across twenty-seven studies reported, there were a total of 1059 embolizations performed on renal AMLs [18–23, 25–29, 31–35, 37–44, 46, 47, 50]. The pooled rate of technical success was calculated to be 95.70% (95% CI: 0.94, 0.97; *I*^*2*^ = 44.15%). Of these, there were sixty-five procedural failures reported (Fig. 6). A total of 163 patients required re-embolization according to twenty-one studies [18–22, 25–30, 33, 36–38, 40, 41, 43, 46, 50] and 16 received further surgical management according to ten of the studies [18, 20, 21, 27, 29, 30, 33, 37, 42, 43]. The data corresponding to peri-operative outcomes is summarized in Table 5.

**Table 5:** Peri-operative outcomes

Study	Total Procedures (*n*)	Technical Success, *n* (%)	Procedural Failures (*n*)	Need for Re-embolization, *n* (%)	Need for Surgical Management, *n* (%)
**Abouelkheir et al.**	38	33 (86.9)	5	4 (10.5)	1 (NR)
**Ahmadov et al.**	48	46 (95.8)	2	7 (NR)	NR
**Anis et al.**	74	68 (91.9)	6	28 (41.1)	4 (5.9)
**Baba et al.**	11	11 (100.0)	0	0 (0.0)	NR
**Bardin et al.**	26	22 (95.6)	4	4 (17.4)	1 (4.3)
**Bishay et al.**	16	16 (100.0)	0	2 (12.0)	NR
**Chaiyasoot et al.**	25	24 (96.0)	1	NR	NR
**Chan et al.**	28	26 (93.0)	2	4 (14.8)	NR
**Chan Park et al.**	NR	NR	NR	NR	NR (1.0)
**Chapman et al.**	20	20 (100.0)	0	1 (4.5)	3 (14.3)
**Chatziioannou et al.**	10	10 (100.0)	0	2 (20.0)	1 (10.0)
**Combes et al.**	30	25 (83.3)	5	3 (10.0)	1 (3.3)
**El Rafei et al.**	30	29 (97.0)	1	4 (NR)	NR
**Farg et al.**	148	136 (91.9)	12	NR	NR
**Han et al.**	NR	NR	NR	2 (NR)	1 (NR)
**Hocquelet et al.**	39	39 (100.0)	0	7 (17.9)	0 (0.0)
**Hongyo et al.**	44	44 (100.0)	0	NR	NR
**Kothary et al.**	30	30 (100.0)	0	NR	NR
**Lee et al.**	119	106 (89.1)	13	4 (NR)	2 (NR)
**Lee et al.**	11	8 (73.0)	3	NR	NR
**Lin et al.**	45	45 (100.0)	0	18 (40.0)	NR
**Nozadze et al.**	NR	NR	NR	10 (20.4)	NR
**Planche et al.**	34	31 (91.2)	3	3 (8.8)	NR
**Prigent et al.**	27	25 (92.6)	2	NR (1.0)	NR
**Ramon et al.**	44	40 (91.0)	4	15 (37.0)	NR (6.0)
**Rimon et al.**	18	17 (94.4)	1	NR	NR
**Rolland et al.**	25	24 (96.0)	1	4 (16.0)	2 (8.0)
**Salık et al.**	13	13 (100.0)	0	NR	NR
**Takebayashi et al.**	NR	NR	NR	NR	NR
**Urbano et al.**	27	27 (100.0)	0	2 (8.0)	NR
**Wang et al.**	79	79 (100.0)	0	39 (49.4)	NR
**Williams et al.**	NR	NR	0	NR	NR

*n *number, *NR *not reported, *SD *standard deviation

### Post-procedural complications and outcomes

Twenty-three studies assessed the post-procedural complications and adverse outcomes associated with TAE for renal AMLs [18–23, 25, 27–29, 35–44, 46, 47, 50]. The analysis revealed a range of post-procedural complications, with the most common being post-embolization syndrome (PES) affecting 299 (47.53%) patients, abscesses occurring in 13 (4.90%) cases, haemorrhage or bleeding events in 10 (5.10%) instances and haematuria in 10 (5.81%) patients. A total of 7 deaths were documented, distributed across 4 of the studies [20, 36, 39, 46]. Additionally, it was reported that 47 patients required use of NSAIDs, and 68 patients required antibiotics in the post-procedural period. A summary of post-procedural outcomes is provided in Table 6.

**Table 6:** Post-operative characteristics

Study	Renal Function Post-TAE, Mean (± SD) mL/min/1.73 m^2^	Serum Creatinine Post-TAE, Mean (± SD) µmol/L	Major Complications, *n* (%)	Minor Complications, *n* (%)	PES (*n*)	Haemorrhage / Bleeding (*n*)	Haematuria (*n*)	Abscess (*n*)	Mortality (*n*)
**Abouelkheir et al.**	NR	61.9 ± 59.8	1 (2.6)	14 (36.8)	12	NR	1	1	0
**Ahmadov et al.**	NR	NR	NR	NR	NR	NR	1	NR	NR
**Anis et al.**	81.9 ± 42.5	NR	13 (19.2)	NR	NR	3	7	1	NR
**Baba et al.**	77.4 ± 24.1	65.4 ± 24.8	NR	NR	NR	NR	NR	NR	NR
**Bardin et al.**	77.0 ± 31.0	NR	3 (13.0)	14 (60.9)	14	2	1	3	1 (unspecified)
**Bishay et al.**	NR	NR	1 (NR)	2 (12.0)	NR	1	NR	NR	NR
**Chaiyasoot et al.**	NR	NR	3 (12.0)	9 (36.0)	5	NR	NR	2	NR
**Chan et al.**	NR	NR	NR	NR	11	1	NR	NR	0
**Chan Park et al.**	NR	NR	NR	NR	NR	NR	NR	NR	NR
**Chapman et al.**	NR	83.0 ± NR	3 (14.3)	5 (23.8)	5	NR	NR	NR	NR
**Chatziioannou et al.**	NR	NR	NR	NR	4	NR	NR	NR	NR
**Combes et al.**	NR	67.0 ± 15.0	NR	NR	9	2	NR	NR	NR
**El Rafei et al.**	NR	NR	NR	NR	19	NR	NR	NR	NR
**Farg et al.**	NR	159.1 ± 37.6	NR	NR	NR	NR	NR	NR	NR
**Han et al.**	NR	NR	NR	NR	NR	NR	NR	NR	NR
**Hocquelet et al.**	NR	70.0 ± 20.0	NR	NR	NR	NR	NR	NR	NR
**Hongyo et al.**	NR	NR	NR	NR	NR	NR	NR	NR	NR
**Kothary et al.**	NR	NR	NR	NR	NR	NR	NR	NR	NR
**Lee et al.**	NR	70.7 ± 14.1	NR	40 (NR)	56	NR	NR	NR	NR
**Lee et al.**	NR	NR	3 (27.3)	NR	7	NR	NR	1	NR
**Lin et al.**	NR	83.6 ± 17.1	NR	NR	29	NR	NR	NR	0
**Nozadze et al.**	80.0 ± 5.9	NR	NR	NR	27	NR	NR	1	3 (unrelated to AML)
**Planche et al.**	NR	83.0 ± NR	2 (6.7)	6 (20.0)	NR	1	NR	2	NR
**Prigent et al.**	NR	73.4 ± 22.6	3 (11.1)	16 (61.5)	15	NR	NR	2	1 (unspecified)
**Ramon et al.**	NR	NR	NR	8 (NR)	5	NR	NR	NR	NR
**Rimon et al.**	NR	NR	NR	NR	2	NR	NR	NR	NR
**Rolland et al.**	NR	68.3 ± 17.0	3 (8.0)	3 (12.0)	1	NR	NR	NR	0
**Salık et al.**	NR	NR	0 (0)	NR	5	NR	NR	NR	0
**Takebayashi et al.**	NR	NR	NR	NR	NR	NR	NR	NR	NR
**Urbano et al.**	NR	NR	NR	2 (7.4)	5	NR	NR	NR	2 (unrelated to AML)
**Wang et al.**	NR	NR	2 (3.0)	5 (NR)	68	NR	NR	NR	0
**Williams et al.**	101.9 ± 29.7	NR	NR	NR	NR	NR	NR	NR	NR

*n *number, *NR *not reported, *PES* post-embolization syndrome, *SD *standard deviation, *TAE *transarterial embolization

## Discussion

Renal AMLs, constituting a spectrum of renal tumours distinguished by the presence of anomalous blood vessels, smooth muscle cells, and adipose tissue, present a multifaceted challenge in clinical practice. Known for their propensity to grow and, in some cases, rupture, these benign lesions demand careful consideration and intervention to prevent life-threatening complications such as haemorrhage from occurring. Among the evolving therapeutic modalities, TAE has emerged as a promising and minimally invasive approach to address the complexities associated with renal AMLs [51]. While individual studies have reported encouraging outcomes, ranging from symptom alleviation to tumour shrinkage, the collective evidence from our single-arm meta-analysis aims to provide a more comprehensive understanding of the role of TAE. Following the inclusion of 32 studies within this meta-analysis, TAE was found to exhibit reduction of tumour size as well as achieve a high degree of technical success. Drawing from a synthesis of these studies, these outcomes demonstrate the procedural proficiency of TAE in achieving its intended outcomes in AML. As such, these findings may provide insight for physicians when selecting the suitable modality of intervention for renal AMLs.

TAE stands as a pivotal intervention in the management of renal AMLs, demonstrating a robust capability for inducing tumour reduction. As such, the success of transcatheter arterial embolization intervention for renal AMLs, hinges on tumour size reduction, which is a parameter consistently validated across the literature [52, 53]. The process begins with the catheterization of the arterial supply to the AML, a step that is meticulously guided by advanced imaging modalities such as CT angiography or magnetic resonance angiography. Once the catheter is appropriately positioned, embolic agents are deployed to occlude the vessels supplying the tumour [54]. In the context of linear dimensions, our meta-analysis explored multiple parameters to identify how TAE led to reduction in tumour size. Our study revealed a mean tumour reduction of 1.848 cm. Tumour reduction of this size reflects the findings from previous meta-analysis and systematic reviews, such as Lin et al. (2019) and Murray et al. (2015), that showed reductions of 2.09 cm and 3.4 cm, respectively [52, 53]. Additionally, from our meta-analysis the mean tumour size reduction in percentage was reported to be 43.298%. These findings are also consistent with Lin et al. (2019) which reported 30% tumour size reduction [53]. Abouelkheir et al. (2022) identified a significant positive correlation between tumour size reduction and the initial tumour size [18]. This is an important consideration for intervention as there is an ongoing debate in the literature as to the correct indication for intervention with TAE according to the size of the renal tumour. The criteria for determining when to treat renal AMLs have been historically unclear. Previous guidelines suggested treatment for AMLs larger than 4 cm, driven by the association between larger AMLs and increased bleeding risk [7–9]. However, recent studies emphasize that size alone should not be the sole factor for intervention. Indicators such as a growth rate exceeding 0.25 cm/year, the presence of TSC, and the manifestation of symptoms (pain or bleeding) have been identified as more nuanced indications for active treatment [48]. Whilst these discussions persist creating the dynamic landscape for intervention, it is clear that TAE leads to reduction in tumour sizes that is also reflected in the literature. Future meta-analyses should systematically compare transcatheter arterial embolization with alternative interventions for renal AMLs. This includes assessing the safety and efficacy of TAE against modalities like radical or partial nephrectomy, nephron-sparing nephrectomy, percutaneous radiofrequency ablation, cryoablation, and mTOR inhibitors. Such comparisons will clarify the relative advantages and disadvantages of each approach, establishing a hierarchy of effectiveness.

The significance of a high technical success rate in TAE for renal AMLs, as demonstrated by our single-arm meta-analysis, underscores the procedure’s reliability and efficiency in achieving its intended outcomes. Our meta-analysis exhibited a success rate of 95.7%, demonstrating the robustness of TAE as a therapeutic intervention. This is consistent with a previous meta-analysis by Murray et al. (2015), which exhibited technical success of 93.3% [52]. Several factors may have contributed to the high technical success rate witnessed in our analysis. Undoubtedly, the procedural expertise and skills of interventional radiologists as well as the collaborative efforts of multidisciplinary teams, plays a pivotal role in achieving this success. Whilst the majority of papers displayed a high degree of technical success, Lee et al. (2009) reported an overall technical success of 73% [35]. This was because their study encountered technical difficulties with tumour vessels being smaller than microcatheters, as well as the vessels being tortuous making the guidance of those catheters more challenging. For this reason, the literature emphasizes the importance of specialized training and experience in navigating the intricacies of renal vasculature, ensuring accurate catheter placement, and achieving effective embolization [54]. Specifically Lee et al. (2009) also discuss the challenges with the placement of these catheters to large proximal branches that supply both the normal parenchyma as well as the tumour [35]. Ensuring optimal preservation of renal function and normal parenchyma in efforts to precisely target and produce adequate embolization of the tumour to slow progression of size and occurrence of bleeding complications remain the mainstay objectives of intervention. Interestingly, Lee et al. (2020) mentions how minimising normal parenchymal injury can be associated with prolonged hospitalisation as compared to the extent of tumour size reduction with TAE [37]. It is also suggested that size alone should not constitute the primary endpoint of treatment and observation of the angiomyogenic volume and components of the tumour can provide a better understanding of the embolization efficacy as evidenced by Planche et al. (2011). Patient selection is also pivotal, necessitating thorough evaluation based on tumour size, location, and overall health status. The ongoing lack of consensus on the choice of embolization agent further complicates treatment decisions for AML. For these reasons, future research should delve into refining embolic agents and delivery systems, exploring their impact on procedural success. Addressing these nuances in patient selection, embolic agents, and defining comprehensive endpoints for treatment efficacy will contribute to a more refined understanding of TAE’s technical success in the management of renal AMLs.

There exists a wide selection of embolic agents to choose from when conducting transcatheter arterial catheterisation for renal AMLs. The extensive variety in the selection of embolic agents across the 32 studies included in our single-arm meta-analysis investigating TAE for renal AMLs highlights the dynamic landscape of this therapeutic intervention. Currently, there is no established consensus regarding the gold standard choice of agent. The choice of embolic agents is often dictated by specific factors, including the size and vascularity of the angiomyolipoma. Ethanol, recognized for its sclerosing properties, is often employed when a more aggressive approach is warranted, particularly for larger or highly vascular tumours. This agent has shown to induce irreversible endothelial damage and tumour necrosis due to its low viscosity and ability to reach tumour capillaries [37]. Polyvinyl Alcohol particles and microspheres, with their mechanical embolization properties, are frequently chosen for their ability to create a physical barrier within the vessels, proving effective in tumours with diverse vascular characteristics. Urbano et al. (2017) mentions that use of ethanol vinyl alcohol (EVOH) has seen high technical success, as seen in many other agents [46]. Within the diverse spectrum of embolic agents utilized in TAE for renal AMLs, ethanol, Polyvinyl Alcohol (PVA) particles and combination of these agents with other materials emerged as the most common regime across the 32 studies included in our single-arm meta-analysis. While our meta-analysis showcases the prevalence of PVA particles, it is important to note that the choice of embolic agent remains influenced by various factors, including tumour characteristics, procedural goals, and clinician preference. As such the presence of a wide selection of agents undoubtedly increases the heterogeneity and warrants the need to establish a consensus regarding the preferred embolic agents for TAE in AML patients.

Our single-arm meta-analysis, revealed a spectrum of post-procedural complications. Notably, Post Embolization Syndrome (PES) emerged as the most common complication encountered in our study with 299 (47.53%) patients reporting this complication. PES, characterized by symptoms such as fever, pain, and nausea, has been consistently reported in the literature as a frequent aftermath of renal embolization procedures [50]. This aligns with findings from previous systematic review and meta-analyses studies such as those conducted by Murray et al. (2015) and Lin et al. (2019), where PES was identified as a prevalent post-embolization complication at a rate of 35.9% and 54.0% [52, 53]. While this minor complication is prevalent, a high proportion of patients improve with their symptoms following management [23]. Preventive (prophylactic) medications for PES were provided before embolization in some studies such as Williams et al. (2006) [49]. Other studies reported routine management of PES with Non-steroidal inflammatory drugs (NSAIDs) after the symptoms had occurred [20], as well as analgesic and antipyretic medications, proved effective [25]. As we contribute to the evolving body of evidence on TAE for renal AMLs, our identification of PES as a primary complication aligns with existing literature, emphasizing the need for clinicians to anticipate and manage this transient yet clinically relevant syndrome in the post-embolization period. In evaluating the safety of TAE for renal AMLs, our meta-analysis revealed that most reported mortalities were not related to the procedure. Specifically, deaths in Nozadze et al. (2021) were attributed to causes unrelated to AML or TAE [36], while Urbano et al. (2017) documented deaths due to stroke and liver failure, which were also not associated with TAE [46]. The causes of death reported by Bardin et al. (2017) and Prigent et al. (2021) were unspecified, leaving them indeterminate in relation to the treatment [20, 39]. These findings suggest a generally low incidence of procedure-related mortality but underscore the need for more detailed and standardized reporting in future studies to better assess and manage the safety profile of TAE.

Our study is not without certain limitations that necessitate careful consideration. One prominent constraint arises from the predominantly retrospective nature of the studies incorporated in our meta-analysis. The absence of prospective as well as randomized clinical trials introduces bias and limits the robustness of our findings. The diverse array of embolization materials used across studies presented another challenge, as different agents were employed alone or in combination, without a clear superiority demonstrated among them, resulting in heterogeneity in outcomes. In this meta-analysis, the ROBINS-I tool was employed to systematically assess the risk of bias in the included non-randomized studies. By evaluating key domains such as confounding, selection bias, and outcome measurement, the ROBINS-I assessment allowed us to critically appraise the internal validity of each study. The results of this risk of bias evaluation informed our confidence in the body of evidence, with studies exhibiting moderate to high risk of bias being carefully considered when interpreting the pooled outcomes for technical success and tumour size reduction. Additionally, we accounted for heterogeneity by using the I² statistic to quantify variability across studies and applied a random-effects model to provide more conservative pooled estimates, considering the moderate to high heterogeneity observed in some outcomes. The studies included encompassed heterogeneous populations which included various presentations and indications for intervention, potentially compromising the generalizability of our findings. Due to this, with the majority of included studies being retrospective, our analysis lacked stratification based on indication, whether for symptomatic relief or prophylactic management. Additionally, the inability to subgroup tumour sizes and the scarcity of available original data further impeded a nuanced analysis of TAE’s therapeutic effects on varying tumour sizes. Subgroup analysis proved difficult due to several limitations. Firstly, the variability in how tumour-related outcomes were reported across studies, including differences in units of measurement and follow-up periods, made it difficult to standardize the data for comparison. Additionally, many studies lacked detailed breakdowns of patient characteristics, such as tumour size or comorbidities, which would have allowed for effective subgroup stratification. Finally, small sample sizes within potential subgroups further hindered the statistical power needed for reliable subgroup analyses, increasing the risk of unreliable conclusions. The absence of a predefined follow-up period introduced variability, with most patients undergoing CT follow-up within 3 months post-TAE, while others had their initial follow-up between 6- and 12-months post-TAE. The reliance on CT scans for follow-up, raises concerns about radiation exposure, suggesting potential benefits from utilizing MRI or ultrasound in long-term follow-up. The absence of a standardized follow-up regime posed a challenge and establishing specific intervals for assessment could enhance the understanding of TAE outcomes over extended periods. Furthermore, there was heterogeneity in the reporting of AML tumours characterised as either the number of lesions, masses or number of patients. This meta-analysis must also account for the differences between centres in how they regulate protocols, choice of equipment, intervention technique, and the experience of operators, therefore also influencing heterogeneity. Overall, despite these limitations, our meta-analysis demonstrates that TAE in AML should be considered as an effective method that can displays a relatively high technical success rate and shows significant tumour size reduction and minimal complications. As such, future studies would benefit by addressing the above limitations when further evaluating the safety and efficacy of TAE in AML patients.

## Conclusions

In conclusion, despite the acknowledged limitations inherent in the predominantly retrospective nature of the studies and the absence of more prospective, randomized clinical trials, our meta-analysis underscores the safety and efficacy of transcatheter arterial embolization (TAE) in renal angiomyolipomas (AMLs). The observed high technical success rate, significant tumour size reduction, and minimal complications support TAE as an effective intervention. Future studies should address the identified limitations to further enhance the understanding of TAE’s safety and efficacy in AML patients.

## Supplementary Information

Supplementary Material 1.

Supplementary Material 2.

Supplementary Material 3.

Supplementary Material 4.

## Data Availability

No datasets were generated or analysed during the current study.

## Abbreviations

AML: Angiomyolipoma
CT: Computed tomography
EVOH: Ethanol vinyl alcohol
LAM: Pulmonary lymphangioleiomyomatosis
MR: Magnetic resonance
NSAID: Non-Steroidal Anti-Inflammatory Drugs
PES: Post-embolization syndrome
PVA: Polyvinyl Alcohol
TAE: Transcatheter arterial embolization
TSC: Tuberous sclerosis complex
