# Supplementary material for: Safety and efficacy of transcatheter arterial embolization in renal angiomyolipomas: a systematic review and meta-analysis
Source: BMC Nephrol. 2025 Mar 31;26:162. doi: 10.1186/s12882-024-03893-4 (PMC11956202; doi:10.1186/s12882-024-03893-4)
Supplement: Supplementary file 1 — Supplementary Material 1. [file 12882_2024_3893_MOESM1_ESM.docx]

**Additional File 1. Actual Search Strategy**

PubMed search

467

Renal AND angiomyolipoma

(("renal"[All Fields] OR "renals"[All Fields]) AND ("angiomyolipoma"[MeSH Terms] OR "angiomyolipoma"[All Fields] OR "angiomyolipomas"[All Fields])

OR

"renal angiomyolipoma*"

("renal angiomyolipoma*"[All Fields])

AND

Embolization

("embol"[All Fields] OR "embolics"[All Fields] OR "embolisations"[All Fields] OR "embolise"[All Fields] OR "embolised"[All Fields] OR "embolising"[All Fields] OR "embolism"[MeSH Terms] OR "embolism"[All Fields] OR "embolic"[All Fields] OR "embolisms"[All Fields] OR "embolization, therapeutic"[MeSH Terms] OR ("embolization"[All Fields] AND "therapeutic"[All Fields]) OR "therapeutic embolization"[All Fields] OR "embolisation"[All Fields] OR "embolization"[All Fields] OR "embolizations"[All Fields] OR "embolize"[All Fields] OR "embolized"[All Fields] OR "embolizes"[All Fields] OR "embolizing"[All Fields])

OR

“artificial embolisation” OR “artificial embolism” OR “artificial embolus” OR “artificial thrombus” OR “embolisation” OR “embolization” OR “embolization, therapeutic” OR “embolotherapy” OR “therapeutic embolization” OR “therapeutic occlusion” OR “transarterial embolization” OR “transarterial embolization OR “transcatheter embolisation” OR “transcatheter embolization” OR “artificial embolization”

("artificial embolisation"[All Fields] OR "artificial embolism"[All Fields] OR (("artificial"[All Fields] OR "artificially"[All Fields]) AND ("embolism"[MeSH Terms] OR "embolism"[All Fields] OR "embolus"[All Fields])) OR "artificial thrombus"[All Fields] OR "embolisation"[All Fields] OR "embolization"[All Fields] OR "embolization therapeutic"[All Fields] OR "embolotherapy"[All Fields] OR "therapeutic embolization"[All Fields] OR "therapeutic occlusion"[All Fields] OR "transarterial embolization"[All Fields] OR "transarterial embolization"[All Fields] OR "transcatheter embolisation"[All Fields] OR "transcatheter embolization"[All Fields] OR "artificial embolization"[All Fields])

EMBASE

Embase 799

Session Results

.......................................................

No. Query Results Results Date

#10. #4 AND #9 799 24 Jul 2023

#9. #5 OR #6 OR #7 OR #8 138,828 24 Jul 2023

#8. artificial AND (embolisation OR embolization) 67,607 24 Jul 2023

#7. therapeutic AND (embolisation OR embolization) 12,484 24 Jul 2023

#6. 'artificial embolisation' OR 'artificial 119,100 24 Jul 2023

embolism' OR 'artificial embolus' OR 'artificial

thrombus' OR 'embolisation' OR 'embolization' OR

'embolization, therapeutic' OR 'embolotherapy' OR

'therapeutic embolization' OR 'therapeutic

occlusion' OR 'transcatheter embolisation' OR

'transcatheter embolization' OR 'artificial

embolization' OR 'transarterial embolization' OR

'transarterial embolization'

#5. 'artificial embolization'/exp 115,904 24 Jul 2023

#4. #1 OR #2 OR #3 5,625 24 Jul 2023

#3. renal AND angiomyolipoma 5,508 24 Jul 2023

#2. 'angio-myolipoma of the kidney' OR 2,723 24 Jul 2023

'angiomyolipoma of the kidney' OR 'kidney

angiomyolipoma' OR 'kidney hemangiomyolipoma' OR

'renal angio-myolipoma' OR 'renal angiomyolipoma'

#1. 'renal angiomyolipoma'/exp 1,001 24 Jul 2023

.......................................................

Cochrane 5

Search Name:

Date Run: 25/07/2023 02:44:26

Comment:

ID Search Hits

#1 MeSH descriptor: [Angiomyolipoma] explode all trees 40

#2 (renal angiomyolipoma):ti,ab,kw (Word variations have been searched) 66

#3 (renal):ti,ab,kw (Word variations have been searched) 68515

#4 (angiomyolipoma):ti,ab,kw (Word variations have been searched) 84

#5 #1 OR #4 84

#6 #3 AND #5 66

#7 #2 OR #6 66

#8 (“artificial embolisation” OR “artificial embolism” OR “artificial embolus” OR “artificial thrombus” OR “embolisation” OR “embolization” OR “embolization, therapeutic” OR “embolotherapy” OR “therapeutic embolization” OR “therapeutic occlusion” OR “transcatheter embolisation” OR “transcatheter embolization” OR “artificial embolization” OR 'transarterial embolization' OR 'transarterial embolisation'):ti,ab,kw (Word variations have been searched) 12314

#9 (artificial AND (embolisation OR embolization)):ti,ab,kw (Word variations have been searched) 814

#10 (therapeutic AND (embolisation OR embolization)):ti,ab,kw (Word variations have been searched) 3587

#11 #8 OR #9 OR #10 12314

#12 #7 AND #11 5

CiNAHL

S1 renal angiomyolipoma*  (437)

S2 renal AND angiomyolipoma*  (573)

S3 S1 OR S2  (573)

S4 artificial AND (embolisation OR embolization)  (76)

S5 therapeutic AND (embolisation OR embolization) (9,705)

S6 “artificial embolisation” OR “artificial embolism” OR “artificial embolus” OR “artificial thrombus” OR “embolisation” OR “embolization” OR “embolization, therapeutic” OR “embolotherapy” OR “therapeutic embolization" OR “therapeutic occlusion” OR “transcatheter embolisation” OR “transcatheter embolization” OR “artificial embolization” OR “transarterial embolization” OR “transarterial embolization”  (15,622)

S7 embolization OR embolization (15,634)

S8 S4 OR S5 OR S6 OR S7 (15,677)

S9 S3 AND S8 (107)

Scopus

( ( TITLE-ABS-KEY ( "renal angiomyolipoma*" ) ) OR ( TITLE-ABS-KEY ( renal AND angiomyolipoma* ) ) ) AND ( TITLE-ABS-KEY ( "artificial embolisation" OR "artificial embolism" OR "artificial embolus" OR "artificial thrombus" OR "embolisation" OR "embolization" OR "embolization, therapeutic" OR "embolotherapy" OR "therapeutic embolization" OR "therapeutic occlusion" OR "transarterial embolization" OR "transarerial embolization" OR "transcatheter embolisation" OR "transcatheter embolization" OR "artificial embolization" ) )

Web of Science

# Web of Science Search Strategy (v0.1)

# Database: Web of Science Core Collection

# Entitlements:

- WOS.IC: 1993 to 2023

- WOS.CCR: 1985 to 2023

- WOS.SCI: 1900 to 2023

- WOS.AHCI: 1975 to 2023

- WOS.BHCI: 2005 to 2023

- WOS.BSCI: 2005 to 2023

- WOS.ESCI: 2005 to 2023

- WOS.ISTP: 1990 to 2023

- WOS.SSCI: 1900 to 2023

- WOS.ISSHP: 1990 to 2023

# Searches:

1: TS=("renal angiomyolipoma*") Date Run: Wed Jul 26 2023 22:05:25 GMT+1000 (Australian Eastern Standard Time) Results: 1970

2: TS=(renal AND angiomyolipoma*) Date Run: Wed Jul 26 2023 22:07:57 GMT+1000 (Australian Eastern Standard Time) Results: 3538

3: #1 OR #2 Date Run: Wed Jul 26 2023 22:08:36 GMT+1000 (Australian Eastern Standard Time) Results: 3538

4: TS=("artificial embolisation" OR "artificial embolism" OR "artificial embolus" OR "artificial thrombus" OR "embolisation" OR "embolization" OR "embolization, therapeutic" OR "embolotherapy" OR "therapeutic embolization" OR "therapeutic occlusion" OR "transarterial embolization" OR "transarerial embolization" OR "transcatheter embolisation" OR "transcatheter embolization" OR "artificial embolization") Date Run: Wed Jul 26 2023 22:12:11 GMT+1000 (Australian Eastern Standard Time) Results: 68166

5: #3 AND #4 Date Run: Wed Jul 26 2023 22:12:47 GMT+1000 (Australian Eastern Standard Time) Results: 434
