# Supplementary material for: Safety and efficacy of transcatheter arterial embolization in renal angiomyolipomas: a systematic review and meta-analysis
Source: BMC Nephrol. 2025 Mar 31;26:162. doi: 10.1186/s12882-024-03893-4 (PMC11956202; doi:10.1186/s12882-024-03893-4)
Supplement: Supplementary file 2 — Supplementary Material 2. [file 12882_2024_3893_MOESM2_ESM.docx]

**Additional File 2. PRISMA Flow Diagram**

Studies from databases/registers **(n = 1065)**

**Identification**

Included studies ongoing **(n = 0)**

Studies awaiting classification **(n = 0)**

Studies included in review **(n = 32)**

Studies excluded **(n = 932)**

Studies not retrieved **(n = 0)**

Studies assessed for eligibility **(n = 85)**

Studies sought for retrieval **(n = 85)**

Studies screened **(n = 1017)**

Studies excluded **(n = 53)**

Language (n = 1)

Wrong setting (n = 5)

Wrong intervention (n = 7)

Wrong study design (n = 18)

Patient Population <10 (n = 7)

Wrong route of administration (n = 1)

Does Not meet Primary Outcomes (n = 14)

References removed **(n = 48)**

Duplicates identified manually (n = 1)

Duplicates identified by Covidence (n = 47)

Marked as ineligible by automation tools (n = 0)

Other reasons (n = )

**Screening**

**Included**
