# Supplementary figures and images for: Safety and efficacy of transcatheter arterial embolization in renal angiomyolipomas: a systematic review and meta-analysis
Source: BMC Nephrol. 2025 Mar 31;26:162. doi: 10.1186/s12882-024-03893-4 (PMC11956202; doi:10.1186/s12882-024-03893-4)

**Additional File 3. ROBINS-1 Risk of Bias and Quality Assessment**


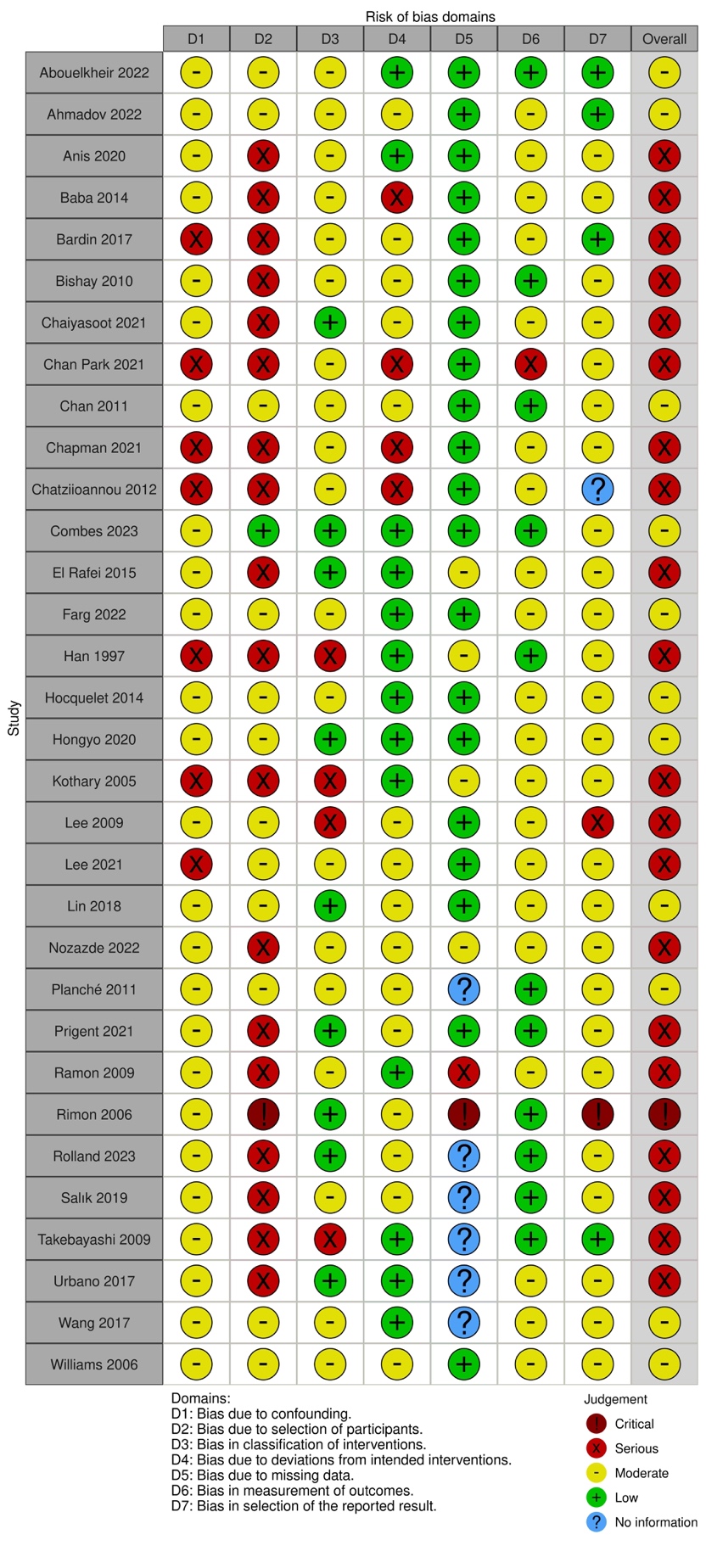


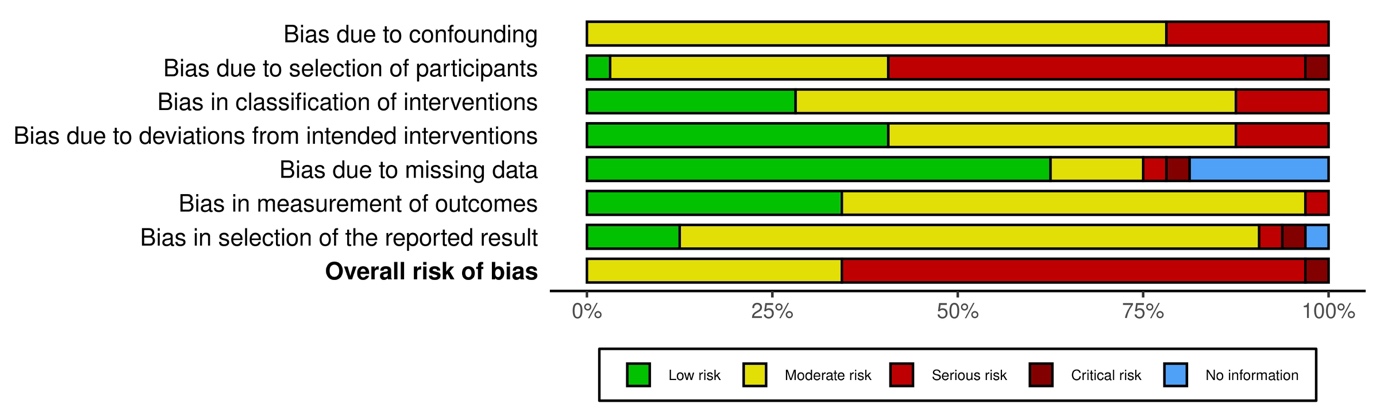

Supplement: Supplementary file 3 — Supplementary Material 3. [file 12882_2024_3893_MOESM3_ESM.docx]

**Additional File 4. Funnel Plot**


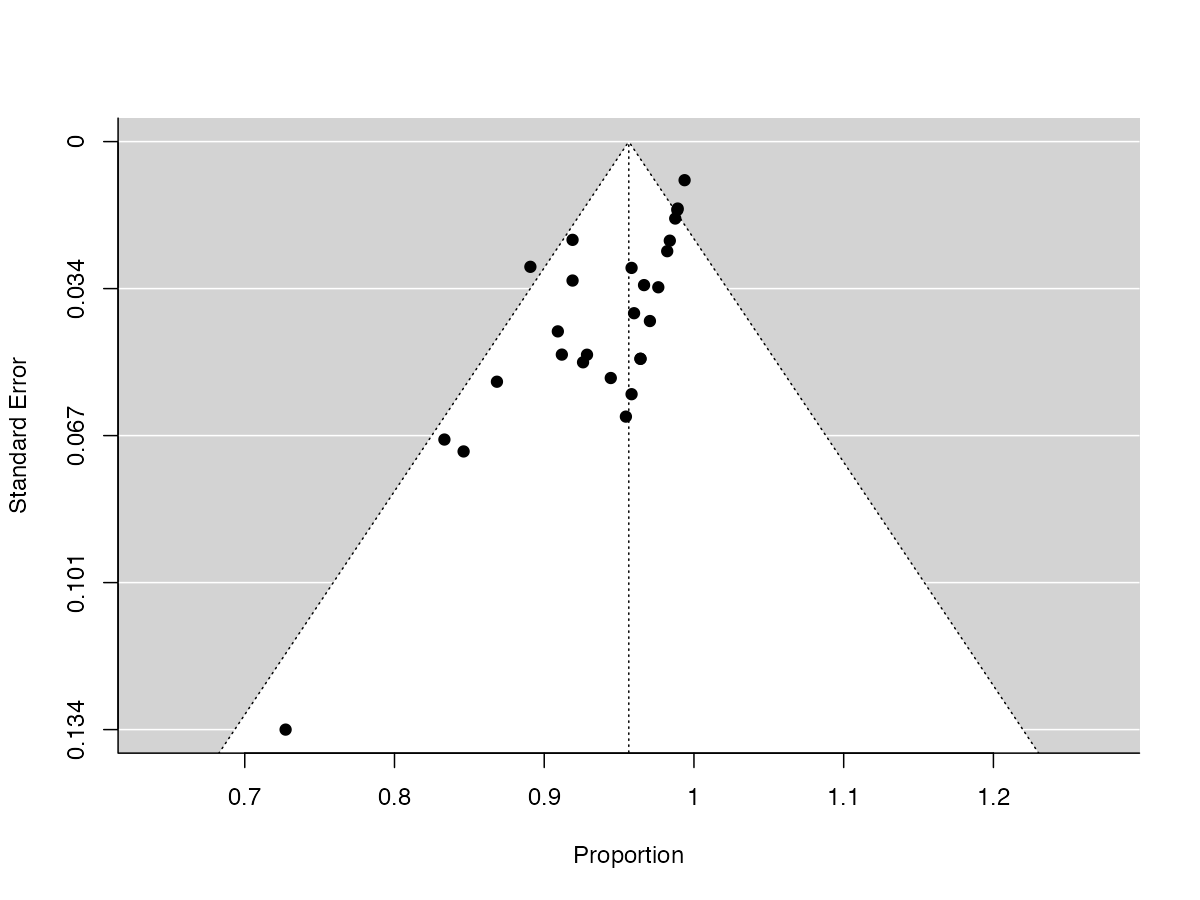
**Pooled Technical Success Rate (%) – Funnel Plot**

Supplement: Supplementary file 4 — Supplementary Material 4. [file 12882_2024_3893_MOESM4_ESM.docx]
